# Synergistic Positive Feedback Mechanisms Underlying Seizure Initiation

**DOI:** 10.1177/15357597221127163

**Published:** 2022-09-27

**Authors:** Andrew J. Trevelyan, Robert T. Graham, R. Ryley Parrish, Neela K. Codadu

**Affiliations:** 1 Newcastle University Biosciences Institute, Medical School, Framlington Place, Newcastle upon Tyne, United Kingdom; 2 Queen Square Institute of Neurology, University College London, United Kingdom; 3 Department of Cell Biology and Physiology, Brigham Young University, Provo, UT, USA

**Keywords:** pyramidal cell, dendritic plateau potential, inhibition, chloride, potassium, ictogenesis

## Abstract

Investigations into seizure initiation, in recent years, have focused almost entirely upon alterations of interneuronal function, chloride homeostasis, and extracellular potassium levels. In contrast, little attention has been directed toward a possible role of dendritic plateau potentials in the actual ictogenic transition, despite a substantial literature dating back 40 years regarding its importance generally in epilepsy. Here, we argue that an increase in dendritic excitability, coordinated across the population of pyramidal cells, is a key stage in ictogenesis.

Understanding how seizures start is an inherently difficult scientific challenge, for multiple reasons. The activity patterns are highly complex and can rapidly spread to involve large areas of the brain, presenting significant difficulties even to identify where a seizure starts. Focusing down too far, recording from individual neurons, runs the risk of failing to see the woods for the trees. While the opposite problem (failing to see the trees at all) is perhaps a better metaphor for most clinical recordings, which are very abstracted and undersampled representations of what we would really like to see, if we aspire to take a scientific approach to managing a particular patient’s problems. All this is made more difficult by the evident variability of epileptic phenotypes.

Recently, considerable insights have been garnered from animal models, aided by the introduction of optogenetic technology. The main debate has focused upon the involvement of GABAergic interneurons^1–5^ and chloride homeostasis^6–12^ in ictogenesis. To understand this debate, it is helpful if we start by describing some notable differences between the models, and then a key point of convergence.

## Differences and Commonalities Between Acute Seizure Models

Early studies of disinhibited cortical networks, induced by blocking GABA_A_ receptors (e.g., using various penicillin derivatives, bicuculline, picrotoxin, or gabazine) provided several key insights, including an enduring model of the paroxysmal depolarizing shift, which recognized the importance of the slow kinetics of voltage-dependent Ca^2+^ channels and NMDA receptors.^13–16^ Another notable result was the demonstration that, in the complete absence of any restraining inhibition, network-wide pathological discharges can be entrained by a single pyramidal cell.^17^ Also noteworthy is that in disinhibition models, the discharges are relatively short-lasting (mean duration ∼1 s), they propagate quickly, and one does not see extended tonic–clonic seizure-like events (SLEs),^18^ which leads us to a critical point that disinhibition models tell us nothing of how GABAergic activity shapes epileptic discharges.

**Figure 1.**
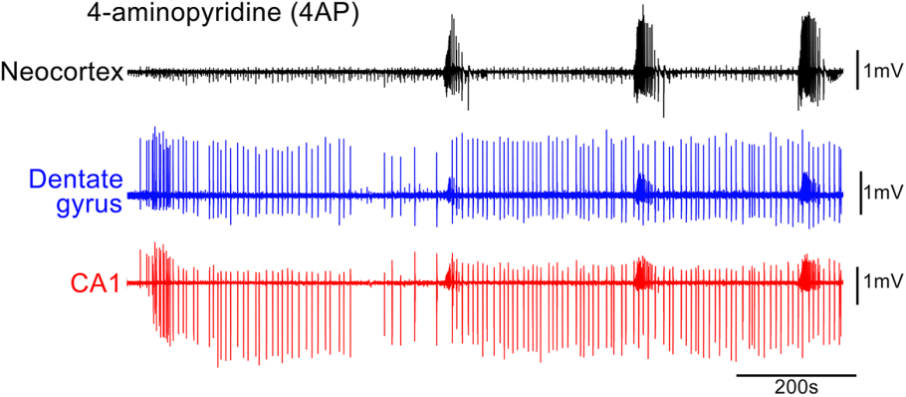
Epileptiform activity in 4-aminopyridine. The earliest pathological activity recorded in a horizontal brain slice prepared from a young adult, wild-type mouse, following application of 100 µM 4-aminopyridine (4AP). Note the prominent early discharges in the hippocampal territories, well in advance of the first seizure-like events (SLEs), that are far more prominent in the neocortex and entorhinal cortex (not shown). Seizure-like events are small in the hippocampus and appear to be secondary generalized from the entorhinal and neocortical areas. See Codadu et al for further details.^20,21^

**Figure 2.**
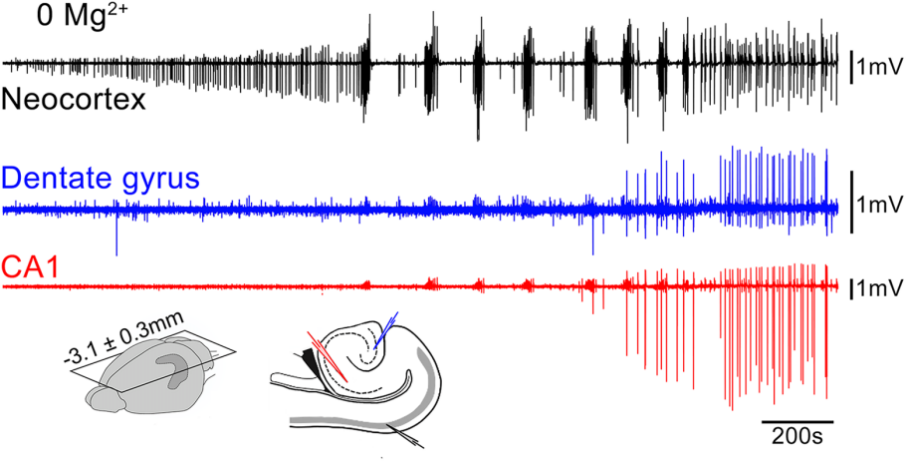
Epileptiform activity in zero Mg^2+^ bathing solution. The earliest pathological activity recorded in a horizontal brain slice prepared from a young adult, wild-type mouse, following wash-out of Mg^2+^ ions. Note the virtual absence of any hippocampal activity, until very late, when it starts to entrain the neocortical activity into what we have termed the late-recurrent discharge pattern. See Codadu et al^20,21^ for further details.

Instead, our current understanding of epileptic GABA involvement stems largely from 3 other experimental paradigms: (1) applying the K^+^ channel blocker, 4-aminopyridine (4AP), which has a disproportionate effect on certain populations of interneurons, inducing intense bursting of these cells (Figure 1); (2) removing Mg^2+^ ions, which enhances synaptic excitation, with only secondary effects on synaptic inhibition (Figure 2); and most recently (3) optogenetics methodology for selective stimulation, or suppression, of particular subclasses of neurons.^19^ In vitro, there are very striking differences in the evolving pattern of pathological network activity in 4AP and 0 Mg^2+^ (see Table 1 ^20,21^; note that another model, induced by raising extracellular potassium, [K^+^]_extra_, shares features with 4AP).

**Table 1.** Key Differences Between 4AP and 0 Mg^2+^ Models.^a^

		4-Aminopyridine	0 Magnesium
Primary pharmacology		Blocks voltage-gated K+ channels	Relieves voltage-dependent NMDA s blockade: enhances Glut neurotransmission
Secondary pharmacology		Effects more apparent in interneurons	Reduces divalent cation shielding—shift in voltage-dependence, enhancing excitability
Primary effect on cell activity		IN burst firing enhanced	Enhances synaptic summation in dendrites
Early interictal activity patterns		Primary IN activity	Glutamatergic, with secondary IN activity
Brain area patterns	Early	Prominent Hipp activity; also NC & EC	NC/EC IIDs; Minimal Hipp involvement
	Intermediate	SLEs in NC, referred to Hipp	SLEs in NC/EC; Minimal Hipp involvement
	Late	Late recurrent discharges; Hipp led, spreading to NC/EC	Late recurrent discharges; Hipp led, spreading to NC/EC
Seizure induction by optogenetic IN activation		Yes	Only in late stage
Electrographic SLE pattern		Low-voltage, fast activity	Hypersynchronous-onset

Abbreviations: 4AP, 4-aminopyridine; EC, entorhinal cortex; Hipp, Hippocampus; IIDs, interictal discharges; IN, interneurons; NC, neocortex; SLEs, seizure-like events.

^a^ See Codadu et al^20,21^ for details; also Levesque et al^22^ and de Curtis and Avoli^23^ about the electrographic SLE patterns at seizure onset, and Chang et al^24^ regarding SLEs being triggered in 0 Mg^2+^ by optogenetic interneuronal activation only once seizure-like activity is well established, and not early in that model.

Much has been made of the fact that, in 4AP, intense optogenetic activation of interneurons generally (channelrhodopsin expressed under the GAD promoter), or of the parvalbumin subpopulation alone, can induce SLEs.^3,19,24-27^ Central to the transition are 2 important ionic redistributions: a rise in intracellular chloride [Cl^−^]_intra_, which in turn can produce large secondary surges also in [K^+^]_extra_, mediated by the potassium-chloride cotransporter, KCC2.^3,28^ The positive shift in the GABAergic reversal potential, E_GABA_, is large enough that GABAergic synaptic barrages can trigger firing—this happens during the clonic stage of seizures.^29^

From these informative optogenetic experiments has arisen the idea that seizures are routinely triggered by interneuronal activation, supported by experimental and human recordings, which show intense interneuronal activity ahead of pyramidal recruitment to seizures,^30-32^ but this would be misleading.

The critical point to understand is that there is a spectrum of GABAergic dysfunction associated with seizures. The 4AP model lies at one end, where a pure GABAergic burst can trigger seizures on the background of raised [Cl^−^]_intra_ and [K^+^]_extra_; at the other end are disinhibition models, when activation of the entire network can arise from stimulation of a single pyramidal cell.^17^ Lying somewhere in between are the 0 Mg^2+^ in vitro model, in vivo models with synaptic inhibition intact, in which seizures are induced by sustained optogenetic stimulation of pyramidal cells,^5^ and more pertinently, sensory-triggered seizures in humans. An important insight from the 0 Mg^2+^ model is that when GABAergic function is preserved (early after Mg^2+^ is removed; late activity patterns have compromised inhibition), even the most intense transient network stimuli do not trigger seizures (although sustained stimulation might), because the cortical microstructure favors inhibition, thereby providing a very effective restraint on activity. This protective inhibitory restraint, however, is rapidly compromised by use, and so the precise ictogenic mechanism depends both upon the level of prior interneuronal activation, and also the size of the glutamatergic drive. Given the various differences between 0 Mg^2+^ and 4AP, of particular note is our finding that a simple optogenetic assay of excitability (testing the postsynaptic response to a focal pyramidal cell activation, triggered using Channelrhodopsin) preempts the onset of seizure like activity in both models.^33^ We describe this next.

## Synergistic Positive-Feedback Forces Underlying Ictogenesis

Cortical function is often discussed in terms of the balance between inhibition and excitation, but a far better framework is the concept of “attractor states,” which derives from the field of state physics.^34^ An attractor is simply a state to which the system converges from various starting points. Where multiple attractors exist, transitions from one to another are dictated by the relative “attractiveness” of each (i.e., the forces drawing the system into each attractor, and how those scale with distance), as well as the landscape of the intervening regions. One may visualize this as a map of energy valleys (the attractors) separated by ridges. The stability of a given attractor is set by negative feedback forces directed back toward its center, correcting any drift away. Forces operating in the opposite direction, by contrast, may be considered positive feedback, propelling the system to a different state. There are good mathematical models of such systems, but these are often rather high-level and abstract, and the difficulty has been to relate these to variables at the molecular or cellular level. A good case in point is the description of seizure initiation in terms of saddle-nodes,^35^ and network resilience^36^ or fragility^37^ (essentially, these 2 latter terms are the reciprocal of each other), which mimic the tipping-point network behavior at a large scale, without specifying the molecular or cellular parameters; we here suggest a model which explains the cellular basis of this critical tipping point, prompted by our optogenetics assay.^33^

We return briefly to Cl^−^ and K^+^ distributions. Chloride is the main permeant anion of GABA_A_ receptors which provide the major synaptic inhibitory drive. Importantly, these receptors are also permeable to bicarbonate ions, and since pH is buffered strongly either side of the membrane, and E_Bicarb_ is positive relative to E_Cl_ (approximately −10 mV and −60 mV respectively), there is a continuous exchange of the 2 ions—inward Cl^−^ and outward HCO_3_^−^ movement—whenever the GABAergic conductance is high, as happens during the repeated bursts of interneuronal activity induced by 4AP. When there is also concurrent excitatory drive, the membrane potential is pushed away from E_Cl_, increasing the driving force for inward Cl^−^ movement, and Cl^−^ influx is greatly accelerated. In distal dendrites, the effect is likely to be exacerbated by the small compartment size and poor diffusion.^38^ The nub is that any significant level of GABAergic activation leads to progressive chloride loading of neurons, which compromises inhibitory function, making further neuronal activation more likely.

Chloride transmembrane distribution is critically coupled to potassium’s, through the action of the cation-chloride cotransporter, KCC2. The K^+^ gradient is fractionally steeper than that of Cl^−^, meaning it is dominant, leading to outward movement of both ions. Chloride-loading reduces the Cl^−^ gradient, further favoring outward K-Cl cotransport. Consequently, after intense GABAergic activation, there occurs a secondary surge in [K^+^]_extra_. The same mechanism underlies the surge in [K^+^]_extra_ that has been recorded ahead of ictal recruitment, or an ictal wavefront, at a time when there is very large GABAergic drive, but relatively little local recruitment of neurons^30^ (meaning that Hodgkin-Huxley K^+^ extrusion is probably quite small). Subsequently, neuronal recruitment exacerbates the rise in [K^+^]_extra_, which may easily then exceed 10 mM during a seizure^39,40^ (Figure 1). A key element of KCC2 function that has not been considered in ictal recruitment is that it operates quite close to its equilibrium, meaning that it may also carry the ions in the opposite direction, with relatively small changes in the concentration gradients of either ion. An interesting consequence of this is that if a subpopulation of neurons experiences an episode of chloride-loading, this leads to a local surge in [K^+^]_extra_ which then causes chloride loading in other neurons; in other words, the effect is coordinated across the network. Glial buffering of [K^+^]_extra_ limits this effect, and so any deficits in glial function, therefore, will make the system more susceptible to these ionic shifts.

**Figure 3.**
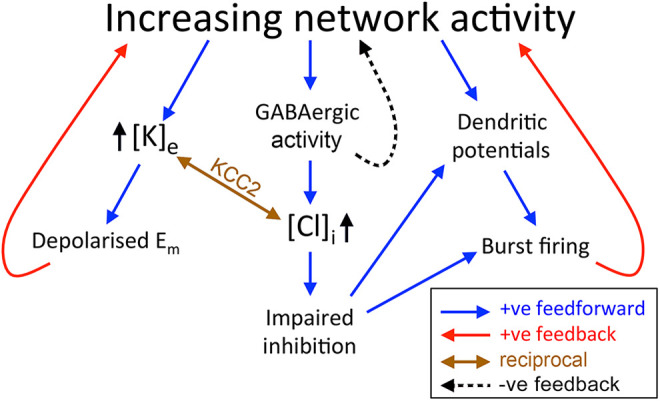
Synergistic positive feedback mechanisms. A schematic of the positive feedback mechanisms, manifest at the cellular level, which contribute to the steep-sided tipping point between physiological and ictal attractor states. An extended discussion of various negative feedback features within cortical networks, that stabilize the normal physiological state, can be found in review by Major et al.^42^

As with raised [Cl^−^]_intra_, raising [K^+^]_extra_ also constitutes positive feedback; both are caused by neuronal activity, and increase the likelihood of further activity. Another positive feedback mechanism that is likely to be critical is the presence of 2 “active” conductances in the dendrites, namely, NMDA receptors and voltage-gated Ca^2+^ channels (VGCCs). Like Na^+^ channels, these are both depolarizing conductances that are opened by depolarization, a positive feedback mechanism that gives rise to action potentials; unlike Na^+^ channels, NMDA receptors and VGCCs open for tens to hundreds of milliseconds, and so give rise to plateau potentials in the dendrites.^13^ This transforms the output of the neuron at the threshold for the dendritic spike. If the level of glutamatergic drive is slightly below this threshold, the neuron may not fire any somatic (Hodgkin-Huxley type) action potentials, or perhaps just one or two. If on the other hand, a dendritic spike is achieved, this generally generates a high frequency burst of firing.^41^ There is thus an all-or-nothing change in the output of neurons, for a fractional increase in the drive. Figure 3 provides a schematic of how these various positive and negative feedback mechanisms interact.

We recently discovered that by monitoring dendritic excitability, using a simple optogenetic stimulation assay, the onset of seizure-like activity in all acute ictogenic models tested is presaged by a sudden step change in the response.^33^ Interestingly, the centrality of dendritic spikes aligns well with computational simulations by Roger Traub and colleagues, from the 1980s, of the paroxysmal depolarizing shift,^14,16,43,44^ incorporating VGCCs, although this work did not focus upon the actual transition into seizures. It is also consistent with a substantial literature associating chronic epileptic phenotypes with changes in expression of various ion channels that influence dendritic excitability, including voltage-gated Ca^2+^ channels,^45^ I_Nap_,^46^ I_A_,^15^ I_h_,^47,48^ and SK-type K^+^ channels^49^ (see also reviews^50,51^). It squares nicely, too, with the observation that multiple antiepileptic drugs appear to act by reducing burst firing in neurons, and that the inhibitory restraint ahead of an ictal wavefront involves intense activation of both parvalbumin and somatostatin interneurons, targeting the soma and the distal dendrites, respectively.^1^ Finally, various studies of neuronal bistability arising from active conductances, such as VGCCs and NMDA receptors, have been proposed to underlie a variety of other network transitions.^42,52-55^ As we have argued elsewhere,^56^ the occurrence of epileptic activity exists only a short step away from normal cortical function.

This focus upon bistable states, arising from all-or-nothing dendritic spikes, has parallels with the 2 levels of activation seen in the thalamus.^57^ When thalamic relay neurons (TRNs) are relatively depolarized, the low-threshold VGCCs are inactivated, and information transfer is relatively precise. In contrast, relatively hyperpolarized TRNs have VGCCs that are activatable, and so are prone to fire intense bursts of action potentials. Feedback loops involving the reticular nucleus can set up powerful oscillations, seen in both sleep spindles and absence seizures.^58^ Despite there being a long-standing consensus regarding the importance of VGCCs and plateau potentials in this thalamic form of seizure, the field has rather lost sight of their importance also in cortical seizures.

In summary, this model of seizure initiation describes how several positive feedback mechanisms—raised [Cl^−^]_intra_, raised [K^+^]_extra_, and the occurrence of dendritic action potentials, which further causes a step increase in the somatic firing output with yet more glutamate being released into the local network—feed into each other. The synergistic nature of these mechanisms creates an especially steep saddle node and explains why the tipping point between normal cortical function and seizure activation can appear to occur with such speed. Importantly, the proximity to the tipping point may be assayed by recording the response to brief stimuli, to provide advance warning of imminent seizures.^33^
